# Preparation and Characterization of Breathable Hemostatic Hydrogel Dressings and Determination of Their Effects on Full-Thickness Defects

**DOI:** 10.3390/polym9120727

**Published:** 2017-12-18

**Authors:** Hong Pan, Daidi Fan, Wei Cao, Chenhui Zhu, Zhiguang Duan, Rongzhan Fu, Xian Li, Xiaoxuan Ma

**Affiliations:** 1 College of Chemistry & Materials Science, Northwest University, Taibai North Road 229, Xi’an 710069, Shaanxi, China; 15229241749@163.com (H.P.); caowei@nwu.edu.cn (W.C.); zch2005@nwu.edu.cn (C.Z.); duan11170@163.com (Z.D.); rongzhanfu@nwu.edu.cn (R.F.); li_xian1214@163.com (X.L.); xiaoxuanma@163.com (X.M.); 2 Shaanxi Key Laboratory of Degradable Biomedical Materials, Department of Chemical Engineering, Northwest University, Taibai North Road 229, Xi’an 710069, Shaanxi, China

**Keywords:** hydrogel-based wound dressing, bacterial barrier activity, hemostasis activity, full-thickness defect

## Abstract

Hydrogel-based wound dressings provide a cooling sensation, a moist environment, and act as a barrier to microbes for wounds. In this study, a series of soft, flexible, porous non-stick hydrogel dressings were prepared through the simple repeated freeze-thawing of a poly(vinyl alcohol), human-like collagen (or and carboxymethyl chitosan) mixed solution rather than chemical cross-linking and Tween80 was added as pore-forming agent for cutaneous wound healing. Some of their physical and chemical properties were characterized. Interestingly, hydrogel PVA-HLC-T80 and PVA-HLC-CS-T80 presented excellent swelling ratios, bacterial barrier activity, moisture vapor permeability, hemostasis activity and biocompatibility. Furthermore, in vivo evaluation of the healing capacity of these two hydrogels was checked by creating a full-thickness wound defect (1.3 cm × 1.3 cm) in rabbit. Macroscopic observation and subsequent hematoxylin eosin staining (H&E) staining and transmission electron microscopy (TEM) analysis at regular time intervals for 18 days revealed that the hydrogels significantly enhanced wound healing by reducing inflammation, promoting granulation tissue formation, collagen deposition and accelerating re-epithelialization. Taken together, the obtained data strongly encourage the use of these multifunctional hydrogels for skin wound dressings.

## 1. Introduction

Skin is a relatively soft tissue, covering the entire external surface of the body. Every year, millions of people suffer skin injuries caused by various factors such as fire, heat, electricity, chemicals, or diseases. The most serious type is full thickness injuries where all the regenerative elements are destroyed and healing occurs from the edges with considerable contraction [1,2]. Thus, a wound dressing that acts as a barrier, that ideally meets the demands of rapid wound closure, promoting wound healing and reducing scar formation is highly required. Hydrogels, produced by chemical or physical crosslinking of soluble polymers [3], are soft and viscoelastic polymeric materials that can retain large amounts of water or biological fluids in their three-dimensional network structure [4,5,6]. Hydrogel based wound dressings possess all the necessary properties that wound healing requires, such as maintaining a moist environment at the wound interface, providing a cooling sensation, allowing gaseous exchange, maintaining a barrier to microorganisms, and allowing wound exudate absorption, hemostatic potential, and biocompatibility [7,8].

Natural hydrogels can be derived from cellulose derivatives, xyloglucan, glycosaminoglycan and collagen [9]. Human-like collagen (HLC) is highly expressed by recombinant Escherichia coli BL21 containing a partial cDNA clone obtained from human collagen mRNA [10,11]. HLC is a promising material which has been successfully explored for the tissue engineering of vascular scaffolds [12], artificial bones [13] and skin tissues [14] as a result of its excellent biocompatibility and low immunogenicity. Unfortunately, human-like collagen alone does not have the mechanical and structural support to perform well after implantation. This has led to research in crosslinking it with other natural or synthetic macromolecules [15]. Poly(vinyl alcohol) (PVA) is a hydrophilic and crystalline polymer with good mechanical properties and biocompatibility. At present, PVA is one of the most frequently used and the oldest synthetic polymers that have been employed in wound dressings, cartilage [16], vascular [17], bone tissue engineering [18] and artificial organs [19]. However, PVA hydrogel has inadequate elasticity, a stiff membrane, and very incomplete hydrophilic characteristics which restrict its use alone as a wound dressing. Therefore, we combined PVA and HLC to prepare composite hydrogels by physical crosslinking, as the physical crosslinking method avoids the presence of crosslinking agents, organic solvents, and chemical reagents thus overcoming the toxicity issue, in comparison to the chemical crosslinking method [20]. When PVA and HLC are combined together, the hydrophilic nature of HLC may improve the wettability and permeability of hydrogels, resulting in culture medium penetration that is favorable for cell adhesion, and PVA is capable of enhancing the mechanical properties of hydrogels.

An ideal hydrogel as a skin dressing should provide a balance between a local pore structure sufficient to retain cell products that form extracellular matrix (ECM) tissue and a global architecture that provides adequate nutrient supply to cells at the central core regions of the scaffold, which means it should hold an interconnected pore structure. Porous hydrogel can be prepared through various means including foaming, pore-forming agent, phase separation [21] and front-line polymerization [22]. We chose Tween80 as the pore-forming agent in this study. Tween80 is a hydrophilic surfactant that can be removed by washing in ultra-pure water after gelation, and the pore size of the hydrogel can be controlled by the amount of Tween80 added. The specific steps are as follows: emulsification foaming of Tween80, freezing ice crystal phase separation, and the removal of Tween80 surfactants. There have been a few reports on the use of Tween80 as a porogen, the use of Tween80 as a porogen for the system composed of PVA, HLC, (or and CS) is first reported in this paper.

Overall, in this study, a series of hydrogel-based wound dressings were developed through repeated freez-thawing method. The macrostructure, microstructure, porosity, mechanical properties, moisture vapor transmission rate, in vitro and in vivo degradation rate, hemolytic properties, and hemostatic performance of the hydrogels were systematically evaluated. The obtained hydrogels had an interconnected pore structure. Moreover, they presented excellent bacterial barrier activity and biocompatibility. Hydrogels PVA-HLC-T80 and PVA-HLC-CS-T80 showed high liquid absorptive capacity, good moisture vapor transmission ability and efficient hemostatic capacity. Furthermore, these two hydrogels were chosen to demonstrate their potential to significantly promote the in vivo wound healing process in a full thickness skin defect model in rabbit, the results of wound closure, H&E analysis and TEM analysis consistently indicated that these two hydrogels could significantly promote the wound healing process.

## 2. Materials and Methods

### 2.1. Materials

Poly(vinyl alcohol) (PVA, Mr 89,000–98,000) and Carboxymethyl chitosan (CMCS) was purchased from Sigma (St. Louis, MO, USA), these two reagents were medical grade. Human-like collagen (HLC, China patent number: ZL01106757.8, Mr = 97,000) was supplied by our laboratory. Roswell Park Memorial Institute 1640 (RPMI 1640) medium and fetal bovine serum (FBS) were obtained from Hyclone (Logan, UT, USA). 3-(4,5-Dimethylthiazol-2-yl)-2,5-diphenyl tetrazolium bromide (MTT) was purchased from Sigma-Aldrich (St. Louis, MO, USA) All aqueous solutions in this experiment were made by using deionized (DI) water. Rabbits as the animal model were obtained from Xi’an Jiaotong University, and all other solvents and reagents were of analytical grade.

### 2.2. Synthesis of the Hydrogels

The hydrogels were prepared by a previously described method (repeated freez-thawing) [23]. The orthogonal test was used to determine the optimal concentration of each component and the number of repeated freez-thawing cycles. A PVA pre-gel solution (10 wt %) was prepared by dissolving 5 g of PVA powder in 50 mL of pure water at a temperature of 90 °C over a period of 2 h. The HLC pre-gel solution (5 wt %) and CMCS pre-gel solution (2.5 wt %) were prepared by dissolving 2.5 g of HLC and 1.25 g of CMCS in 50 mL of pure water respectively. The preparation of the poly(vinyl alcohol)-human collagen (PVA-HLC) hydrogel was carried out by mixing the PVA and HLC solution at a volume ratio of 2:1, then decanting the mixture into a customized square mold (with a square length of 40 mm) and freezing for 20 h at −20 °C, then thawed for 4 h at room temperature, this process was repeated twice. The PVA-HLC-T80 hydrogel was prepared by the same process except that Tween80 was added to the mixed solution at a volume ratio of 1:25 before pouring into the mold. For PVA-HLC-CS and PVA-HLC-CS-T80, the volume ratio of PVA, HLC and CS solution was 5:2:2, and Tween80 was added as described above. The obtained hydrogels were then soaked in ultrapure water for three days with magnetic stirring to remove the Tween80, the water was refreshed every two hours.

### 2.3. Microstructure Analysis of Lyophilized Hydrogels

Lyophilized samples were immersed in liquid nitrogen for 3 min and then brokenoff to detect the fracture surface. All prepared samples were gold coated and analyzed using scanning electron microscope (Hitachi S-570, Tokyo, Japan).

### 2.4. Fourier Transform Infrared (FT-IR) Spectroscopy and Thermal Gravimetric Analysis (TGA)

Different components of the lyophilized hydrogel systems were verified by FT-IR. Infrared spectra of the specimen powders, namely PVA, HLC, CS and the hydrogels as well as the Tween80 solution was recorded using an FT-IR spectrophotometer (Thermo Fisher Scientific, Waltham, MA, USA). The samples were triturated with KBr at a ratio of 1:100 and pressed to form pelleted samples for FT-IR spectroscopic analysis at 500–4000 cm^−1^.

The thermogravimetric analyzer (TGA) (Netzsch, Selb, Germany) was used to analyze the chemical structure of various hydrogels at different temperatures. Nitrogen was used as the protective gas, at a flow rate of 80 cm^3^/min. The 50 mg sample was placed into the platinum crucible and heated from room temperature to 600 °C at a rate of 2 °C/min.

### 2.5. Density and Porosity of the Lyophilized Hydrogels

The weight of the lyophilized hydrogels was measured by a one over ten-thousand analytical balance, and the length, width and height were measured by Vernier calipers, the density of the hydrogels was calculated by the formula below

(1) $$d=\frac{m}{a\times b\times c}$$

where d represents the density (g/cm^3^). m represents the mass (g). a, b and c represent the length, width and height respectively (cm).

Porosity of the lyophilized hydrogels was evaluated using an alcohol displacement method [24]. Briefly, the hydrogels were immersed in absolute ethanol until saturated. The hydrogels were weighed before and after the immersion in alcohol. The porosity was calculated using the formula

(2) $$P=\frac{W2-W1}{\rho V1}\times 100\%$$

where W1 and W2 indicate the weight of the hydrogels before and after immersion in alcohol, respectively. V1 is the volume of the hydrogel before immersion in alcohol; and ρ is a constant (the density of alcohol). All samples were triplicated in the experiment.

### 2.6. Swelling Kinetics

The lyophilized hydrogels were immersed in deionized water that placed in a 37 °C water bath and removed at regular time intervals. The excess water on the surface was wiped off, and the hydrogels were weighed until equilibrium was reached. The water uptake capacity (Wu) was determined as follows:

(3) $$\mathrm{Wu}=\frac{\mathrm{Mt}-\mathrm{Mg}}{\mathrm{Mg}}\times 100\%$$

where Wu is the water uptake capacity. Mt and Mg are the weight at regular time intervals and the xerogel, respectively.

The water holding performance of the hydrogels was determined by placing the swollen-balanced hydrogels at room temperature until a constant weight was reached, and the hydrogels were weighed at regular time intervals.

### 2.7. Tensile Property of the Hydrogels

Tensile tests were performed in accordance with ASTM D638 with a crosshead speed of 1.0 mm/min [25]. The hydrogels were cut into total length of 5.5 cm, breadth of 1.5 cm and for the gauge length of 2 cm. The samples were equilibrated with 0.1 M phosphate buffer solution (PBS) (pH 7.4), and the elasticity was investigated by applying a load of 200 g at a cross-head speed of 10 mm/min. The ultimate tensile strength of the material was determined from the stress (kPa) versus strain (%) curve and the elongation length at break was also recorded.

### 2.8. Moisture Vapor Transmission Rate

The water vapor transmission rate of the hydrogels was measured according to the ASTM method, E 96–95 [26]. The test was carried out at a temperature of (21 ± 2) °C and a relative humidity of (60 ± 15)%. The device of this test was prepared in our laboratory. A measuring cup, which was corrosionresistant, impermeable and airtight, had water added to it so that the gap between the liquid level and the placed sample was 5 ± 1 mm and sealed the cup with the hydrogel dressing (with a thickness of 1.5~2 mm). The effective area of the sample was measured (57.2 cm^2^), the total weight of the cup, sample, and distilled water was weighed and recorded as W1. Then, the equipment was placed in a drying oven or incubator. The same measuring cup, exposed in air, was used as the control. Five parallels were made for each sample. After 18 to 24 h, the equipment was removed from the oven or incubator, the test time (T) was recorded, the equipment was immediately re-weighed and recorded as W2. The moisture vapor transmission rate (MVTR) was calculated by the following formula:

(4) X=(W1−W2)×1000×24/T

where: X represents the moisture vapor transmission rate (MVTR) (g·m^−2^·24 h). W1 represents the mass of the container, sample and liquid (g). W2 represents quality of containers, samples, and liquids after test period (g). T represents the test period (h).

### 2.9. In Vitro Degradation Rate of the Hydrogels

The in vitro degradation rate of the lyophilized gels was measured using stimulated body fluids (SBF). After being weighed and sterilized by Co60 irradiation (24 h, 10 kGy), the dried gel samples were immersed in tubes containing 4 mL of fresh SBF, and the tubes were kept static at 37 °C in an incubator. The samples were withdrawn from the SBF solution and then rinsed with ultra-pure water three times after soaking at different times. The dry weight was measured after lyophilization. The weight loss was calculated as:

(5) WL=(W0−W1)/W0 ×100%

where W0 and W1 are the weights of the gel before and after soaking, respectively.

### 2.10. In Vitro Hemolytic Activity Test of the Hydrogels

A hemolysis activity assay was conducted to evaluate the blood compatibility of the lyophilized hydrogels. In this study, an extract of the hydrogel was used to determine the hemolysis of the material. To prepare the extract, the hydrogels were sterilized by Co60 irradiation, cut into small pieces and placed in tubes with fresh culture medium added at 0.1 mL/g at 37 ± 0.5 °C for 72 h, note that the whole process should be operated at a clean bench. Distilled water was used as the positive control and normal saline as the negative control.

The specific test process is as follows: 5 mL of blood was taken from a healthy rabbit ear artery into a anticoagulant tube, then the tube was shaken quickly until the solution was mixed evenly. Four mL of this anticoagulant blood was taken out and 5 mL of 0.9% normal saline solution was added to prepare a fresh dilution of rabbit blood. Then, 5 mL of the four extracts, distilled water and normal saline solution respectively was taken, and 0.1 mL of the rabbit blood dilution was added to each tube and slowly mixed until even. Then the tubes were placed in a 37 °C constant temperature water bath for 1 h. After the water bath, all the tubes were centrifuged at 4 °C (1500 rpm, 5 min) and the supernatant was carefully aspirated. Their absorbance at 545 nm was measured with a spectrophotometer.

### 2.11. Bacterial Barrier Properties of the Hydrogels

The bacterial barrier property of the hydrogels was determined according to the ASTM F1608:2016 standard. Staphylococcus aureus or *E. coli* were lawn cultured on a replicate organism detection and counting (RODAC) contact plate filled with nutrient agar medium. After incubation for 24 h, the sterilized hydrogel sheets were placed on the surface of the plate, then another RODAC contact plate with nutrient agar medium was placed on the hydrogel samples and suppressed with a weight to create constant pressure on the materials. After incubation for 24 h, 48 h, and 72 h, the RODAC contact plate on the top was removed and cultured at 30 °C for 24 h to observe the presence of any colony.

### 2.12. Hemostatic Property of the Hydrogel

The hemostatic property of the lyophilized hydrogels was evaluated as described in [27]. A bleeding ear artery rabbit model was employed and all animal studies were performed in compliance with guidelines set by national regulations and were approved by the local animal experiments ethics committee (Protocol number: NWU201705235). The rabbit was anesthetized by injecting 3 wt % pentobarbital sodium (1 mL/kg) and fixed on a surgical corkboard. The hair on back ear was shaved and the ear was sterilized with 75% alcohol. A pre-weighted filter paper on a polyurethane film was placed beneath the ear. A 1 cm × 1 cm wound including the ear artery was created, and the pre-weighted dressing was applied over the bleeding area after free hemorrhage for 5 s and constant pressure (200 g) was exerted. The dressing was removed and weighted until blood was absolutely coagulated and the hemostatic time recorded. Total blood loss was also evaluated by the difference value between the weights of dressing and filter paper before and after the hemostatic assay.

### 2.13. Cytotoxicity Testing of Hydrogels

The cytotoxicity of the lyophilized hydrogel extracts was assessed using an MTT based on colorimetry where viable cells can reduce water-soluble MTT to a colored formazan product. The hydrogels were sterilized by Co60 irradiation and then placed into tubes with fresh culture medium added at 0.1 mL/g, cultured at 37 ± 0.5 °C for 72 h to achieve hydrogel extracts. L929 cells were cultured at a density of 1.0 × 10^4^ cells/mL on 96-well plates (100 μL/well) in a CO_2_ (5%) incubator at 37 °C. After incubation for 24 h, the extracts were added to 96-well plates (100 μL/well) in a CO_2_ (5%) incubator at 37 °C. After incubation for 24 h, 72 h, and 120 h, 10 μL of MTT solution was added to each well, and the cultures were incubated at 37 °C for 4 h. The post incubation media containing MTT was removed, and the purple formazan crystals that formed were dissolved by incubating the hyrogel in 1.5 mL of dimethyl sulfoxide (DMSO) for 15−20 min at room temperature (RT) with constant shaking. The absorbance of the solution was measured at 490 nm.

### 2.14. In Vitro Biocompatibility of the Hydrogel

Fibroblast (L929) cell lines were cultured in 1640 media supplemented with 1% penicillinstreptomycin and 10% fetal bovine serum (FBS) in an incubator at 37 °C, 5% CO_2_. The hydrogels (1~2 mm thickness and 10 mm diameter) were sterilized by Co60 irradiation (72 h, 10 kGy) and then they was washed 2−3 times with 1× PBS with 10−15 min incubation for each wash. Finally, the hydrogels were incubated in cell culture media for 24 h. To test the biocompatibility of the hydrogel, 1 × 10^5^ of cells were seeded on each hydrogel sample. The media was refreshed every day.

#### 2.14.1. MTT Assay

Cell viability was measured at regular time intervals by MTT assay. The hydrogels were sterilized by Co60 irradiation, then placed into tubes with fresh culture medium added at 0.1 mL/g, and cultured at 37 ± 0.5 °C for 72 h to achieve the hydrogel extracts. L929 cells were cultured at a density of 1.0 × 10^4^ cells/mL on 96-well plates (100 μL/well) in a CO_2_ (5%) incubator at 37 °C. After incubation for 24 h, the extracts were added to 96-well plates (100 μL/well) in a CO_2_ (5%) incubator at 37 °C. After incubation for 24 h, 72 h and 120 h, 10 μL of MTT solution was added to each well, the cultures were incubated at 37 °C for 4 h. The post incubation media containing MTT was removed, and the purple formazan crystals that formed were dissolved by incubating the hydrogel in 1.5 mL of dimethyl sulfoxide (DMSO) for 15−20 min at RT with constant shaking. The absorbance of the solution was measured at 490 nm.

#### 2.14.2. Microscopic Analysis

The cells were allowed to grow for a period of 7 days in the hydrogel. These were analyzed by fluorescent staining. One mm thick sections were selected and stained with acridine orange/ethidium bromide (AO/EB) staining solution followed by detection with an inverted fluorescence microscope.

### 2.15. In Vivo Histocompatibility Evaluation

All experiments were performed in compliance with the relevant laws and institutional guidelines, and conducted with the approval of the Institute Animal Ethics Committee (ethical approval code NWU201705235). This study was supported by the Shaanxi Key Laboratory of Degradable Biomedical Materials and Northwest University. The histocompatibility of hydrogels implanted into the body was measured at an immune system response level by hematoxylin and eosin (H&E) staining and transmission electron microscopy (TEM) analysis.

#### 2.15.1. Implantation of Hydrogels

The biocompatibility of the PVA-HLC, PVA-HLC-Tween80, PVA-HLC-CS, and PVA-HLC-CS-Tween80 was assessed in vivo by implanting the shaved and disinfected lyophilized hydrogels into rabbits. All of the rabbits were quarantined and allowed free access to food and water, but were not given antibiotics for a week before surgery. The animals were raised in a relative humidity of 50–60%, a controlled temperature of 20–22 °C and a 12 h light-dark cycle. Dorsal hairs of rabbits were removed by depilation so that a naked region was obtained for the operation. The rabbits’ backs were implanted with eight hydrogel sheets (0.5 cm × 0.5 cm), each of the four hydrogel dressings was repeated twice. After 2, 4, 6 and 8 weeks, the rabbits were depilated again and sacrificed. The gross appearance of the implanted sites was photographed with a digital camera (Nikon TE2000-U), and the gel color, redness, edema, fester, vessels, and fiber capsule were assessed. The implant/tissue constructs were harvested and cut in half; one half was used for H&E staining and the other half for TEM.

#### 2.15.2. H&E Staining and TEM Analysis

In this analysis, the implant/tissue constructs harvested from the rabbits were immediately fixed in 10% neutral buffered formalin and left overnight. The constructs were rinsed with 0.1 M PBS (pH 7.4) for several minutes to remove residual formalin, then successively placed in a series of ethanol baths from 70 to 100% ethanol before immersion in a mixture of ethanol and xylene (1:1 *v*/*v*%), and xylene for 30 min each to ensure that the tissues were transparent. Next, the constructs were immersed in liquid wax overnight until the tissues were filled, then carefully embedded in epoxy resin. The blocks were sliced on a Leica RM2016 diamond saw microtome (Leica Instruments Ltd., Wetzlar, Germany) with a blade thickness of 5 μm and assembled on coated slides.

For TEM analysis, the ultrathin sections (50 to 70 nm) were stained with 4% uranyl acetate and 0.5% lead citrate and observed by transmission electron microscopy (TEM) (HITACHI, H-600, Tokyo, Japan).

### 2.16. In Vivo Studies in Rabbit Animal Model

Three-month old healthy female white New Zealand rabbits weighing 2−3 kg were used for experiments.

#### 2.16.1. Surgical Procedure

Before starting the surgery, rabbits were anaesthetized by giving intravenous injection with 3% pentobarbital (20 mg/kg). Thereafter, an electric shaver was used to remove hair from the dorsal area and skin was sterilized using 70% ethanol. According to the principle that the least number of animals were used, eight full thickness square defects were created (1.5 cm × 1.5 cm) on each rabbit and every two symmetrical wounds taken as a group. The first two groups of wounds were treated with the PVA-HLC-Tween80 lyophilized hydrogel and PVA-HLC-CS-Tween80 hydrogel, respectively. For comparison, the third group was treated with commercially available alginate dressings and the last group was left untreated (control). Each of these dressings was implanted on ten wounds for each time point (6th, 12th, and 18th day after surgery). After the dressings were implanted, the wound area was covered with cotton gauze which was fixed with polyurethane film. After surgery, rabbits were housed individually in the cages. To evaluate repair, rabbits were sacrificed by giving intravenous injection of air after the 6th, 12th, and 18th day of recovery. Specimens with whole wound areas were collected for further analysis.

#### 2.16.2. Macroscopic and Histological Evaluation

After sacrificing the rabbits, the skin samples of the wound site were collected and fixed in 10% formalin saline. Furthermore, samples were dehydrated by treating them with ethanol gradient. Thereafter, they were embedded in paraffin. Sections of 5 μm were cut and mounted on glass slides. For histological analysis, prepared sections were stained with hematoxylin and eosin. Samples of all the animals were analyzed for wound healing. There were two wounds per rabbit and five rabbits per condition making it 10 wounds per time point per condition. For each parameter, entire histological samples were analyzed with 10 fields taken under consideration. For comparison, the histology of normal rabbit skin was also performed.

#### 2.16.3. Transmission Electron Microscope Analysis

To determine if the different treatments given generate any inflammatory response in rabbits, TEM analysis was undertaken. For this, the ultrathin sections (50 to 70 nm) were stained with 4% uranyl acetate and 0.5% lead citrate and observed by transmission electron microscopy (TEM) (HITACHI, H-600, Tokyo, Japan).

### 2.17. Statistical Analysis

The data were collected in a Microsoft Excel 2007 database, all the experiments were carried out in triplicate, and the results are represented as average ± SD of 3−6 samples used per experiment using Origin 7.0 software. The student’s *t*-test was performed to compare statistical significance between the two groups. A value of *p* < 0.05 was considered to be statistically significant, “*” and “**” represent the *p*-value < 0.05 and *p*-value < 0.01, respectively.

## 3. Results

### 3.1. Macrostructure and Microstructure of the Hydrogels

**Figure 1:**
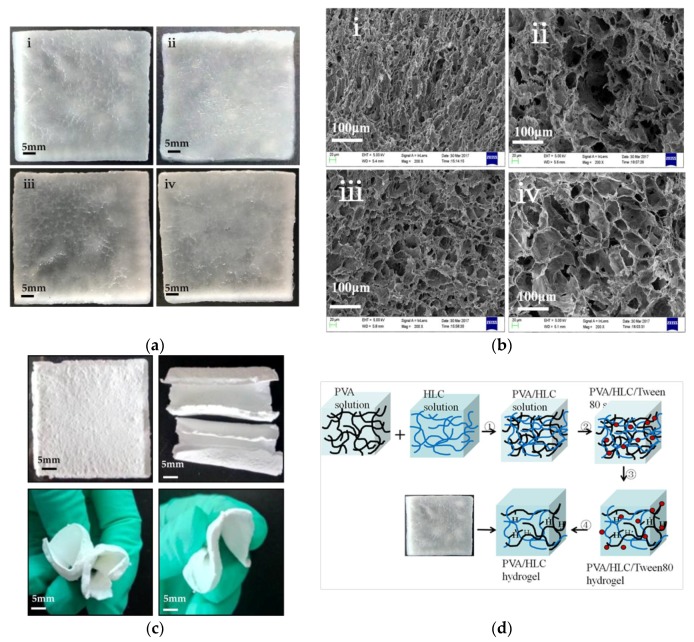
Morphology of the hydrogels. (**a**) morphology of wet hydrogels (**i**) PVA-HLC (**ii**)PVA-HLC-T80 (**iii**) PVA-HLC-CS (iv) PVA-HLC-CS-T80; (**b**) SEM photo of the hydrogels (**i**) PVA-HLC (**ii**) PVA-HLC-T80 (**iii**) PVA-HLC-CS (**iv**) PVA-HLC-CS-T80; (**c**) The hydrogels with Tween80 kept soft after lyophilization; (**d**) The formation process of PVA-HLC-T80 ① Solution blending process ② Addition of Tween80 ③ Repeated freeze-thawing process ④ The process of removing Tween80. PVA: Poly(vinyl alcohol). HLC: Human-like collagen. CS: Chitosan. T80: Tween80.

Four hydrogels were obtained, and they were named PVA-HLC, PVA-HLC-T80, PVA-HLC-CS, PVA-HLC-CS-T80 respectively according to their forming process, and their specific composition are shown in Table 1. Figure 1d shows the forming process of PVA-HLC-T80 as an example to describe the preparation of these hydrogel dressings.

The resulting hydrogel dressings before lyophilization were all translucent (Figure 1a), soft, elastic, porous, non-adhesive sheets with a smooth surface which had a well interconnected network of pores (Figure 1b). The upper and lower surfaces of the hydrogel dressings were asymmetrical with a loose lower surface which had a pore size of 100–150 μm tight upper surface with a pore size no more than 20 μm.

The hydrogels PVA-HLC-T80 and PVA-HLC-CS-T80 looked like sponges, and had large pore size range lying between 100 and 150 μm as seen in the SEM images while the pore size of PVA-HLC and PVA-HLC-CS was much smaller (20–50 μm). The large macroporous network of pores in the hydrogel will provide adequate space for cells to grow and allows the media and other nutrients to reach them freely without clogging the pores.

It was noticeable that, after lyophilization, the hydrogels PVA-HLC-T80 and PVA-HLC-CS-T80 remained soft, and could be bent to any shape to fit the wound and promote the healing process, as shown in Figure 1c.

### 3.2. FT-IR Spectroscopic and TGA Analysis

**Figure 2:**
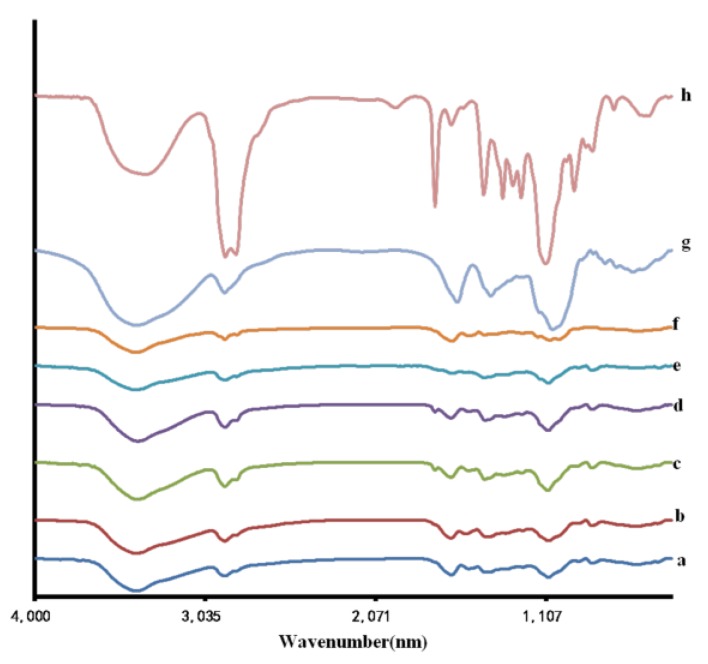
FT-IR spectra of different hydrogels and their components. (a) PVA-HLC; (b) PVA-HLC-T80; (c) PVA-HLC-CS; (d) PVA-HLC-CS-T80; (e) PVA; (f) HLC; (g) CS; (h) Tween80. PVA: Poly(vinyl alcohol). HLC: Human-like collagen. CS: Chitosan T80: Tween80.

The components of the hydrogel systems were assessed using Fourier transform infrared spectroscopy (FT-IR). The spectra obtained for the hydrogels PVA-HLC, PVA-HLC-T80, PVA-HLC-CS, PVA-HLC-CS-T80 and their components are displayed in Figure 2.

**Figure 3:**
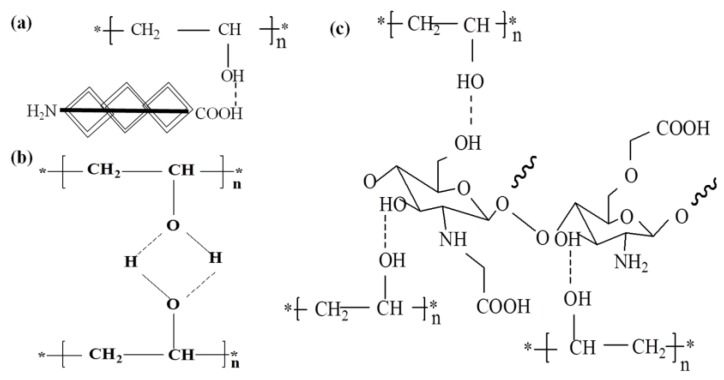
Crosslinking mechanism of the hydrogels. (**a**) Hydrogen bonds between PVA and HLC; (**b**) Intermolecular hydrogen bonds of PVA; (**c**) Hydrogen bonds between PVA and CS. PVA: Poly(vinyl alcohol). HLC: Human-like collagen. CS: Chitosan.

Figure 2 shows the Fourier transform infrared spectroscopy of the four hydrogels and their components. It can be seen that when compared with the various components, there no new chemical bond was generated during the gel process, indicating that these hydrogels were formed by physical cross-linking, there was no copolymerization or polycondensation reaction between the components. During the process of freez-thawing, part of the PVA formed intermolecular hydrogen bonds (Figure 3b) and the crystalline region was produced. The hydrogen bonding effect was also reacted between PVA and HLC molecules (Figure 3a), PVA and CMCS molecules (Figure 3c).

**Figure 4:**
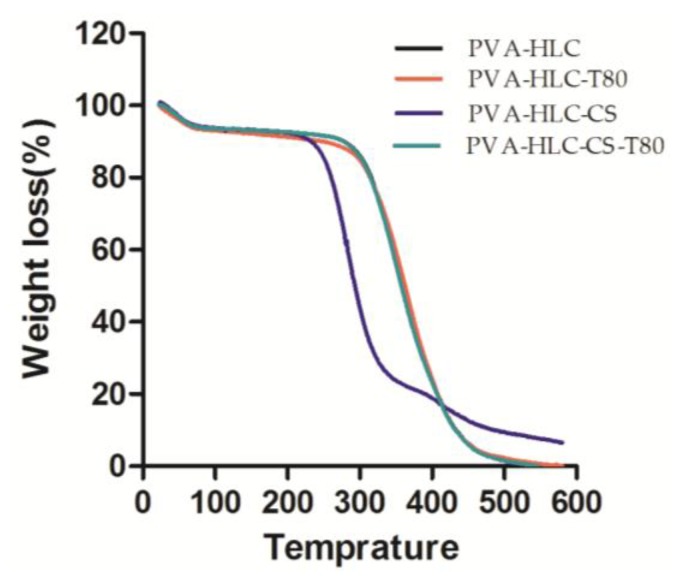
Thermal Gravimetric Analysis (TGA) curves of different hydrogels.

Thermogravimetric analysis TGA involves the measurement of changes in the weights of the samples instrumentally, over a specific range of temperature. The effects of molecular weight and the number of different components added to the thermal stabilities of dry hydrogels were evaluated in the temperature range of 20–600 °C. Results of the thermogravimetric analyses of the synthesized hydrogels are presented in Figure 4. It shows two main decomposition steps for the hydrogels. The initial decomposition temperature was from 20 °C to 100 °C, the corresponding weight losses of PVA-HLC, PVA-HLC-T80, PVA-HLC-CS and PVA-HLC-CS-T80 were 6.43%, 7.01%, 6.25% and 6.38% respectively. The weight loss at this stage was mainly due to the loss of bound water in the hydrogel. The second decomposition temperature range for the hydrogel PVA-HLC, PVA-HLC-T80 and PVA-HLC-CS-T80 was from 250 °C to 430 °C, 200 °C to 330 °C for PVA-HLC-CS. Weight loss at this stage was caused by the thermal decomposition of different components. When the temperature reached 500 °C, the residual weights of PVA-HLC, PVA-HLC-T80, PVA-HLC-CS and PVA-HLC-CS-T80 were 1.56, 2.32, 9.45 and 1.47 respectively, these are the weight value of carbon and ash after decomposition. The difference in degradation temperature was most likely due to the different amounts of PVA and HLC, as well as the pores in different hydrogels, which all contributed to the thermal stability of hydrogels [28].

### 3.3. Density and Porosity of the Hydrogels

Hydrogels PVA-HLC-T80 and PVA-HLC-CS-T80 had much smaller density (0.045~0.055 g·cm^−3^) than PVA-HLC and PVA-HLC-CS due to their porous sponge-like structure, as shown in Table 2.

Porosity is an important aspect of scaffold and biomaterials engineering [29]. The high porosity of the dressings could benefit by absorbing exudate from the wound surface [30]. Furthermore, the presence of a large volume of porosity is also beneficial for the transfer of nutrients and oxygen to the cells attached to the dressings. From Table 1, we can see that the porosity of hydrogel PVA-HLC-T80 and PVA-HLC-CS-T80 ranged between 80~90%, far higher than that of the PVA-HLC and PVA-HLC-CS (39.88% and 38.20% respectively). Thus, the high porosity of PVA-HLC-T80 and PVA-HLC-CS-T80 has great benefit on the transmission of oxygen and nutrients for the wound area of the skin, which then promotes the wound healing process.

### 3.4. Swelling Kinetics

One of the most important properties of a hydrogel is to maintain its state when it absorbs and holds a lot of water. The water in the hydrogel determines the permeability of the hydrogel to nutrients and, to a certain extent, its ability to export cellular products from the hydrogel. Therefore, a desirable dressing material should reach a swelling and deswelling equilibrium in a short time.

**Figure 5:**
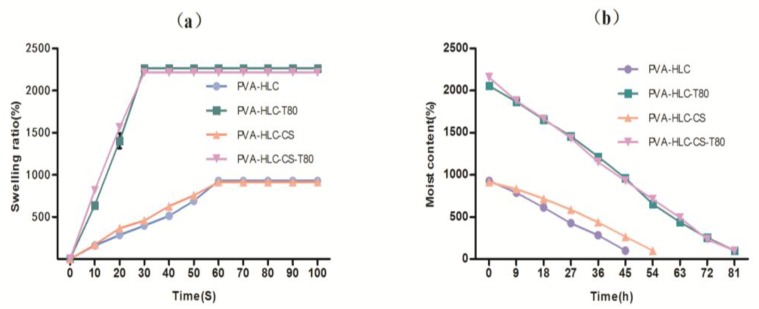
Swelling ratio and water retention capacity of the hydrogel dressings. (**a**) Swelling ratio of different hydrogels; (**b**) Water retention time of different hydrogels.

From Figure 5, we can see that hydrogels PVA-HLC-T80 and PVA-HLC-CS-T80 achieved swelling equilibrium within 30 s, and the water uptake capacity was found to be 2263.8% and 2216.1% at equilibrium, while PVA-HLC and PVA-HLC-CS took one min to achieve swelling equilibrium, and their water uptake capacity was smaller than 1000%. As for water retention time, PVA-HLC-T80 and PVA-HLC-CS-T80 took 81 h to return to dry state while PVA-HLC and PVA-HLC-CS needed 45 h and 54 h, respectively. The results revealed that hydrogel PVA-HLC-T80 and PVA-HLC-CS-T80 could quickly absorb a large amount of wound exudate, and hold water in the hydrogels for a relatively long time, thus providing a wet recovery environment for the wound, which meaning that they can be used as dressings for wounds which have a large amount of exudate.

### 3.5. Tensile Property of Hydrogels

**Figure 6:**
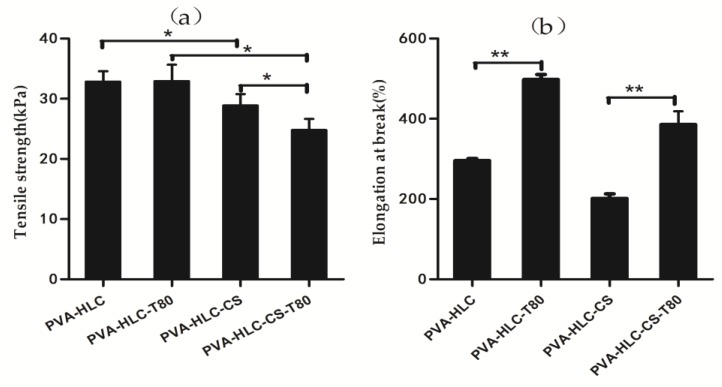
Tensile properties of the hydrogel dressings. (**a**) Tensile strength of different hydrogels; (**b**) Elongation at break of different hydrogels. Where “*” and “**” represent the *p*-value < 0.05 and *p*-value < 0.01, respectively.

The tensile property of a dressing material is an important factor that affects their practical use in the protection of a wounded area. In this study, the tensile strength and value of elongation-at break of the prepared dressings were obtained, and the corresponding data are displayed in Figure 6. From Figure 6a, we can see that the tensile strength of the four hydrogels showed a gradual decline trend. The tensile strength of PVA-HLC was 32.84 kPa, which was the largest one, followed by PVA-HLC-T80 (31.45 kPa) and PVA-HLC-CS (27.63 kPa), and the smallest value appeared in hydrogel PVA-HLC-CS-T80, which was only 25.55 kPa. All the hydrogels showed the regular behavior of a flexible elastomer. With the addition of Tween80, the modulus values of the hydrogels decreased slightly. Meanwhile, the addition of Tween80 almost doubled the value of the elongational break. Furthermore, the hydrogels thus formed did not show any sign of deformation upon stretching.

The elongation value at break indicates the flexibility of the dressings (Figure 6b). Compared with PVA-HLC and PVA-HLC-CS, the values of the elongation at break of PVA-HLC-T80 and PVA-HLC-CS-T80 increased by 65% and 100% respectively, which indicate that these two dressings can be stretched into any shape, which is more suitable for wound without distortion.

### 3.6. Moisture Vapour Transmission Rate (MVTR)

Researchers reported that the regular lost-water from normal skin is ~250 g·m^−2^·d^−1^ at 35 °C, this amount of water loss extensively increase to 5000 g·m^−2^·d^−1^ based on the nature of wound [31]. As a dressing, good moisture permeability can provide a moist environment for wound recovery, while reasonable moisture permeability can prevent excessive loss of exudate which causes dehydration. Queen [24] reported that an ideal dressing material should have a 2000~2500 g·m^−2^·d^−1^ of the water vapor transmission rate.

It can be seen from Table 3 that the water vapor transmission rate of PVA-HLC-T80(2398.4 g·m^−2^·d^−1^) and PVA-HLC-CS-T80 (2887.4 g·m^−2^·d^−1^) showed a great improvement when compared with that of PVA-HLC and PVA-HLC-CS which were 923.2 g·m^−2^·d^−1^ and 921.1 g·m^−2^·d^−1^ respectively, indicating that PVA-HLC-T80 and PVA-HLC-CS-T80 were more ideal as wound dressings.

### 3.7. In Vitro Degradation Rate of the Hydrogel Dressing

**Figure 7:**
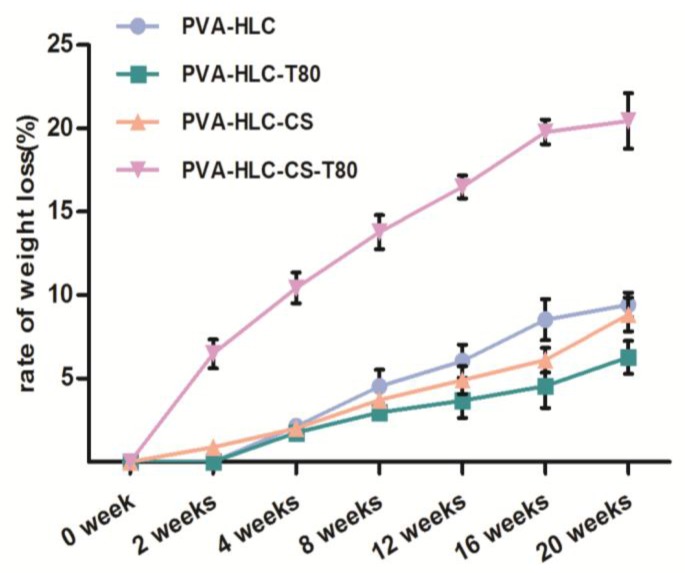
In vitro degradation rate of different hydrogels.

Figure 7 shows the degradation rate of the hydrogels by the simulated body fluid. It can be clearly seen that after 20 weeks of SBF degradation, the weight loss increased 20% for PVA-HLC-CS-T80 and less than 10% for the other hydrogels, indicating that the degradation rates of these hydrogels were all very slow. This may be due to the main component of these hydrogels being PVA. However, PVA is difficult to degrade by simulated body fluid and the diffusion of water into PVA matrices is faster than degradation. Thus, the hydrogels begin to swell prior to degradation. The degradation process of hydrogels is divided into two stages. When the hydrogels were immersed into the SBF solution, water permeated into the matrices making them swell. Accompanying the hydration process, bond cleavage and degradation occurred [32].

PVA-HLC-CS-T80 revealed the fastest degradation, partly attributed to the addition of carboxymethyl CS and HLC, which could be degraded by SBF slowly, partly due to the porous structure of PVA-HLC-CS-T80, where SBF could enter into the interior pores to speed up degradation. SBF in contact with the CS and HLC in the hydrogels, resulted in the hydrolysis reaction of CS and HLC, where chains were degraded, the relative molecular mass gradually decreased, before finally becoming hydrolyzed into oligosaccharides and amino acids, respectively.

### 3.8. Hemolytic Activity Test of the Hydrogels

**Figure 8:**
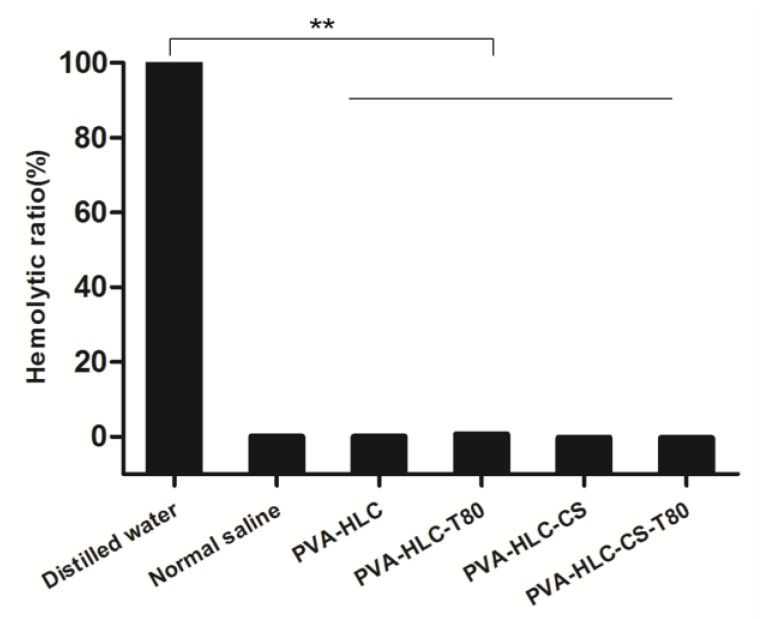
Hemolysis ratio of the hydrogel dressings. Where “**” represent the *p*-value < 0.01, respectively.

The blood compatibility of the hydrogels was further evaluated by testing the hemolytic activity of the hydrogels. The results are shown in Figure 8, where the hemolysis ratio of the four hydrogel extracts was the same or smaller than the negative normal saline group, which demonstrated that these hydrogels had good blood compatibility.

### 3.9. Bacterial Barrier Properties

Bacterial infections can increase the formation of exudate and delay the wound healing process [33]. An ideal wound dressing should block the bacteria infection and provide a comfortable environment for the wound, thus promoting the wound healing process by reducing the number of pathogens and the inflammatory response of the wound site. The bacterial barrier property of the hydrogels against both *E. coli* (Gram-negative bacterium) and *S. aureus* (Gram-positive bacterium) was evaluated by the method described in YY/T 0471.5-2017. After contacting with the hydrogels for 24 h, 48 h, and 72 h, no colonies were found on all the five RODAC contact plates of each hydrogel, which meant that all of the hydrogels could hold excellent bacterial barrier properties. The bacterial barrier property of these materials is mainly due to the interconnected porous network of the hydrogels as the accumulation of layers of holes can block bacteria getting close to the wound surface, thus preventing bacteria from penetrating through the dressings and touching the wound surface, causing infection.

### 3.10. Hemostasis Activity

**Figure 9:**
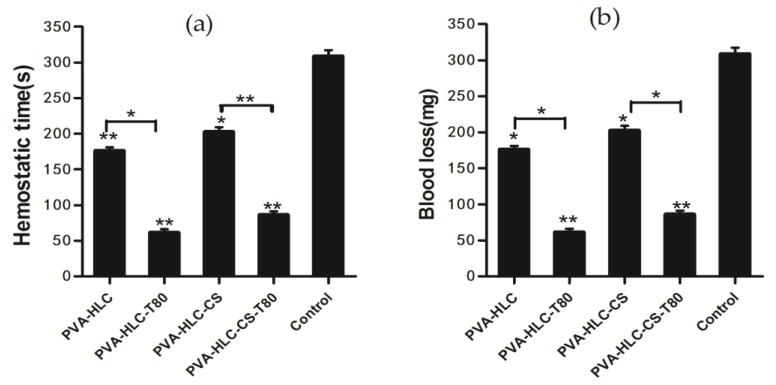
Hemostatic activity of different hydrogels. (**a**) Hemostatic time; (**b**) Blood loss. Where “*” and “**” represent the *p*-value < 0.05 and *p*-value < 0.01, respectively.

Figure 9 shows the hemostatic efficacy of different hydrogels evaluated by the rabbit ear artery hemorrhage model. Figure 9a shows the mean bleeding time and Figure 9b reveals the blood loss of different hydrogels applied on the wound. The bleeding time of PVA-HLC-T80 was 55.25 s, which was the shortest one, followed by PVA-HLC-CS-T80 (80.25 s), PVA-HLC (176.25 s), PVA-HLC-CS (202.50 s) and control groups (202.50 s). The blood loss of the PVA-HLC-T80 and PVA-HLC-CS-T80 were 21.30 mg and 49.28 mg respectively. In contrast, the blood loss of PVA-HLC, PVA-HLC-CS was 129.00 mg and 163.84 mg respectively, but were all less than the control groups, at 320.25 mg. Above all, these hydrogels had the ability to stop the bleeding. When compared with the control groups, the hydrogel PVA-HLC-T80 and PVA-HLC-CS-T80 had excellent hemostasis ability.

The hemostatic behavior of the hydrogels relates to the hydrophilic property of the hydrogels, since the water absorption of PVA-HLC-T80 was the highest, the porous structure could provide many hydrophilic groups and improve the water absorption of the hydrogels [34], where a large amount of water in the blood was absorbed and the density of coagulation factors increased rapidly. As a result, the hydrogels PVA-HLC-T80 and PVA-HLC-CS-T80 can be applied to heavy wound bleeding due to their strong absorption and quickly clotting properties.

### 3.11. Cytotoxicity of Hydrogels

**Figure 10:**
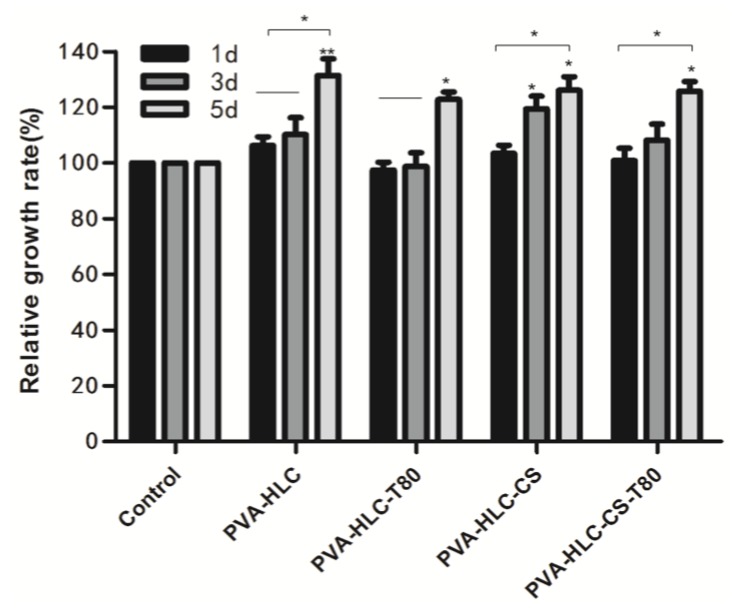
Relative growth rate of cells cultured by different hydrogel extracts for different time intervals. Where “*” and “**” represent the *p*-value < 0.05 and *p*-value < 0.01, respectively.

An ideal soft wound dressing should not release toxic products or produce adverse reactions, which can be evaluated through an in vitro cytotoxic test. Incubation of cells with extracts of the four hydrogels for one, three and five days resulted in significant differences (Figure 10). We can see that the hydrogels PVA-HLC-T80 and PVA-HLC-CS-T80 inhibited the proliferation of L929 cells slightly on the first day, and the relative growth rate were 97.476% and 99.846% respectively, whereas the inhibition disappeared on the fifth day. On the whole, the density of cells cultivated with extracts of the four hydrogels increased after one, three and five days. Compared with PVA-HLC-T80 and PVA-HLC-CS-T80, PVA-HLC and PVA-HLC-CS triggered a significant increase in the number of L929 cells on all five days (*p* < 0.05). According to the ISO standard (ISO10993.12-2005), the toxicity of the four hydrogels was classified as grade 0 with the exception of the PVA-HLC-T80 and PVA-HLC-CS-T80 extracts, which was grade 1 on the first day. The results indicated their potential application as hydrogel composites for specific uses such as moisture wound dressing.

**Figure 11:**
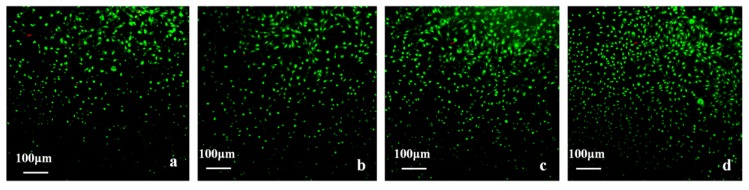
AO/EB staining of the morphology of L929 cells cultured by hydrogel extracts for 3 days, extracts of (**a**) PVA-HLC; (**b**) PVA-HLC-T80 (**c**) PVA-HLC-CS (**d**) PVA-HLC-CS-T80. AO/EB: Acridine orange/ Ethidium bromide. PVA: Poly(vinyl alcohol). HLC: Human-like collagen. CS: Chitosan. T80: Tween80.

It can be seen from Figure 11, that after being cultured for three days, a large number of cells grew in the hydrogel extracts with normal morphology and strong cell viability, and the density of cells was consistent with the result of the MTT assay.

### 3.12. In Vitro Biocompatibility of the Hydrogel

For cytocompatibility analysis, L929 cells were cultured directly on the hydrogels.

#### 3.12.1. Cell Proliferation

**Figure 12:**
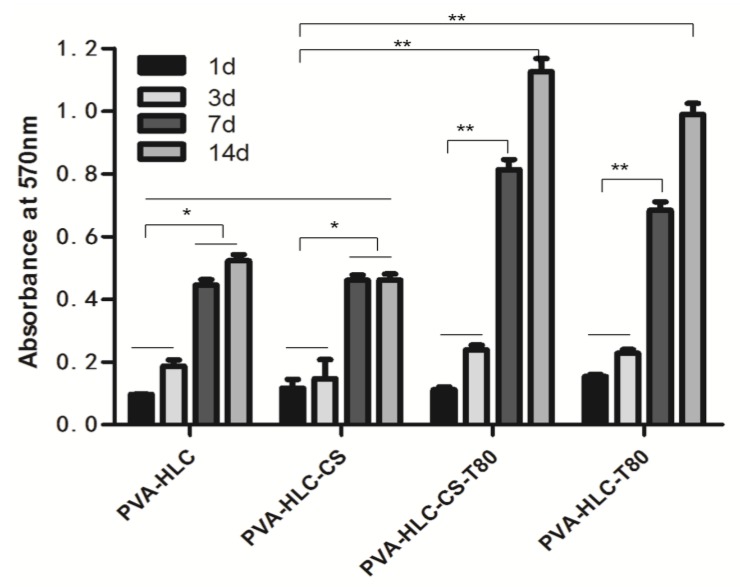
Cell proliferation on different hydrogel dressings. Where “*” and “**” represent the *p*-value < 0.05 and *p*-value < 0.01, respectively.

As shown in Figure 12, the number of cells attached to all scaffolds increased during the entire culture period. On day 1, there was no significant difference in the cell number among the four hydrogels except for PVA-HLC-CS-T80, which was 1.5 times higher than the others. However, after three days, the density of cells in PVA-HLC-T80 and PVA-HLC-CS-T80 was higher (*p* < 0.05) than that in PVA-HLC and PVA-HLC-CS. Even after seven days and 15 days, the number of cells in the two hydrogels with Tween80 were significantly higher (*p* < 0.05) than the others. This may have been due to the hydrogels PVA-HLC-T80 and PVA-HLC-CS-T80 having a suitable cell growth pore size, where cells can grow and proliferate freely, while cells can only grow on the limited surface of the other two hydrogels thus hindering their growth and proliferation.

#### 3.12.2. Cell Viability

**Figure 13:**
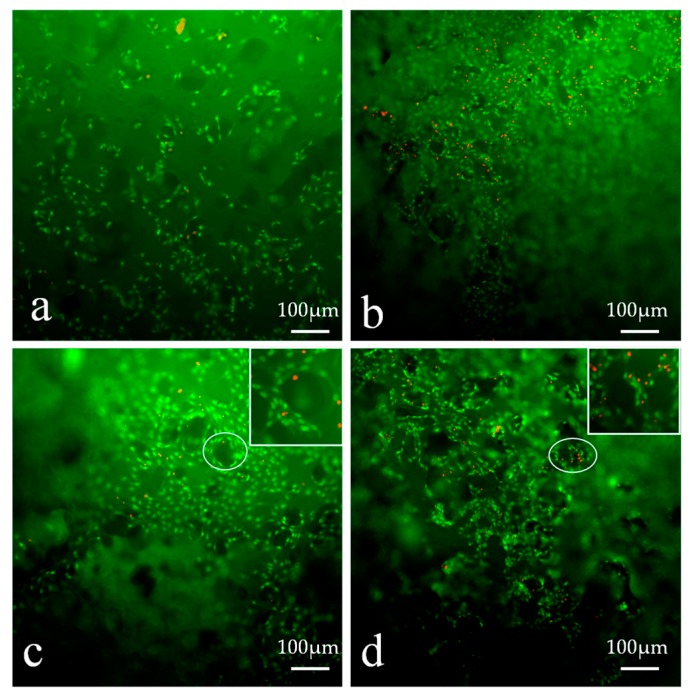
Cell morphology and viability on different hydrogel dressings, (**a**–**d**) represent PVA-HLC, PVA-HLC-T80, PVA-HLC-CS, PVA-HLC-CS-T80 respectively.

The hydrogels, together with the cells grown on them, were stained with an AO/EB staining kit and observed using inverted fluorescence microscopy. The stained images of the fibroblast cells seeded in scaffolds on the seventh day are shown in Figure 13. The fibroblast cells grew and covered the surface of the hydrogels and grew into the pore structure in the surface. Cells presented a strong green fluorescence signal, indicating good viability with any of the hydrogel samples. The images demonstrated that L929 cell adhesion was higher on PVA-HLC-T80 (Figure 13b) and PVA-HLC-CS-T80 (Figure 13d). Indeed, surface hydrophilicity, hydration and swelling as well as surface roughness are considered as important for cell attachment [35], which is why more cells adhered, grew and proliferated on the hydrogels PVA-HLC-T80 and PVA-HLC-CS-T80.

Compared with PVA-HLC-T80, the density of cells on PVA-HLC-CS-T80 was much larger, perhaps due to the addition of CS providing more nutrients for cell growth thus providing the potential to offer the fibroblast cells a more favorable growth environment.

### 3.13. In Vivo Histocompatibility Evaluation

The in vivo toxicity and biocompatibility of the hydrogels are important factors for researchers when evaluating biomaterials. In this study, square hydrogel sheets (6 mm × 6 mm × 1.5 mm) were implanted into the experimental rabbit, when after two, four, six and eight weeks, the hydrogels and rabbit tissues were taken and then assessed by H&E staining and transmission electron microscopy analysis (tissues taken at second and eighth week).

#### 3.13.1. Macroscopic Observation of Implanted Hydrogels and Surrounding Tissue

**Figure 14:**
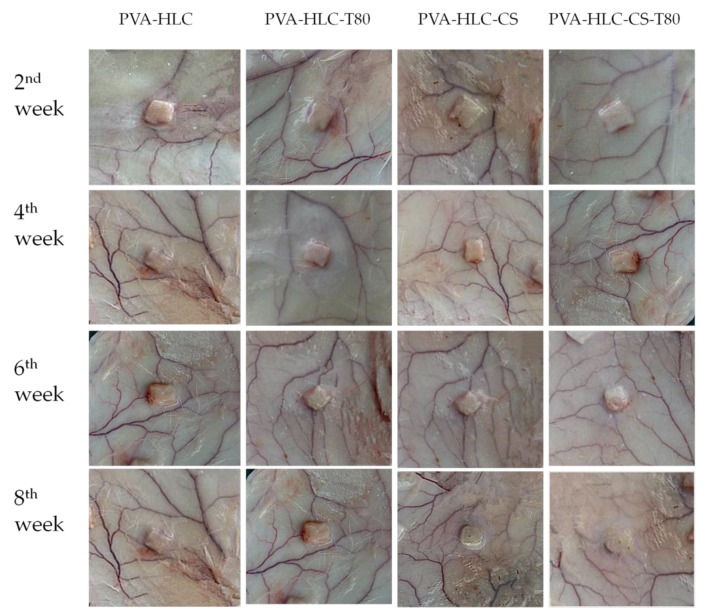
Morphology of different hydrogel implanted in rabbit after different weeks.

After implantation, the hydrogels formed regularly shaped skin protrusions and showed no significant degradation after eight weeks. Around the implantation site, we observed no abnormalities had developed in the tissue (Figure 14). The slight streak of blood observed at the second week indicated that the hydrogels were subjected to phagocytosis by inflammatory cells. However, there were no obvious symptoms after four weeks, indicating that the hydrogels gradually adapted to the tissue surrounding the implantation site. Moreover, no vascular proliferation or redness appeared at the implantation site, which implies the better histocompatibility of the hydrogels. After eight weeks, the PVA-HLC and PVA-HLC-T80 hydrogels maintained their integrity, while PVA-HLC-CS and PVA-HLC-CS-T80 showed a slight degradation as the corners disappeared and the hydrogels changed into a cylindrical shape. Furthermore, the inflammation almost disappeared.

#### 3.13.2. H&E Staining Analysis

**Figure 15:**
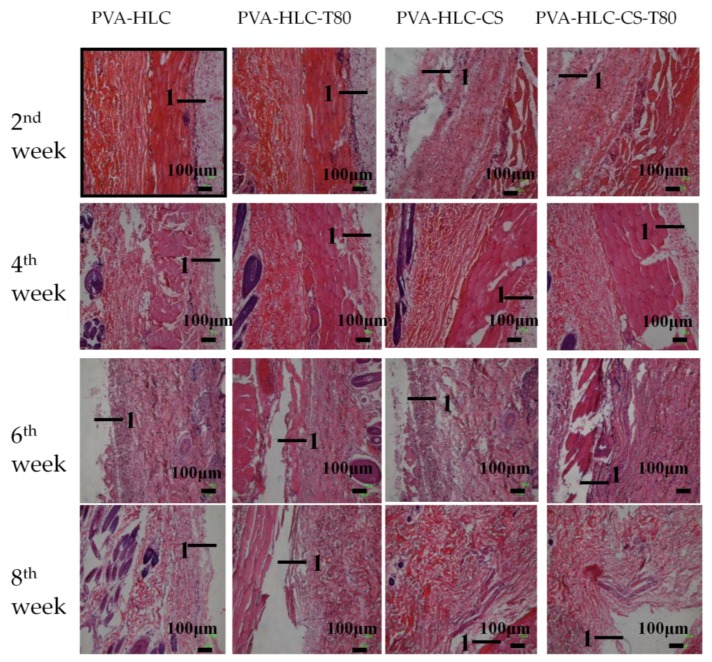
H&E photo of different hydrogels implanted in rabbits after different weeks, “1” represents the position of the hydrogels.

The histological response to the implantation of the hydrogels at two, four, six and eight weeks was analyzed by H&E staining. As shown in Figure 15, the hydrogels that had been implanted subcutaneously into the rabbit for two weeks showed inflammatory cells around the tissue, and all the hydrogels did not begin to degrade. In addition, the tissue and the materials were easily separated. Four weeks later, the inflammatory cells alleviated as the hydrogels adapted to the surrounding tissues and the PVA-HLC-CS and PVA-HLC-CS-T80 hydrogels revealed signs of degradation. Six weeks later, we could not see any inflammatory cells. Finally, after eight weeks, the inflammatory reaction had totally vanished.

The difference in the degradation of the four hydrogels in vivo may be due to the presence of certain enzymes such as collagenase and the free radicals produced by the inflammation reaction, which are involved in the process of degradation. The main component of these hydrogels is PVA, which is difficult to hydrolyze, or become enzymatic in vivo. Compared with hydrogel PVA-HLC and PVA-HLC-T80, which were formed of PVA, HLC, hydrogel PVA-HLC-CS and PVA-HLC-CS-T80 were made of PVA, HLC, and CMCS, where HLC can be degraded by collagenase and CS can be hydrolyzed by body liquids.

#### 3.13.3. TEM Analysis

**Figure 16:**
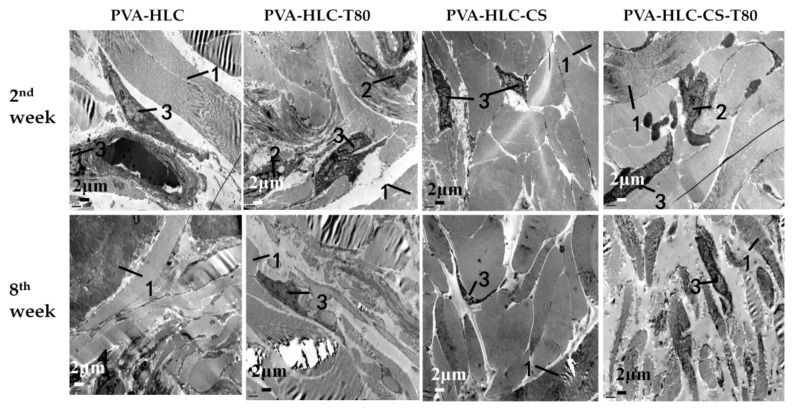
TEM photo of different hydrogels implanted in rabbits after different weeks, 1, 2, 3 and 4 represent the hydrogel, macrophage, lymphocyte and neutrophile granulocyte respectively.

Four types of hydrogels were implanted in the rabbit subcutaneous, where after a period of time after post the implantation, tissues around the material were fixed and detected by a transmission electron microscope to observe the change in cells, tissues, and various organs, as shown in Figure 16.

After implantation for two weeks, a large number of collagen fibers could be seen between the fibroblasts, moreover, there was a considerable number of macrophages and a small number of lymphocytes that existed in the surrounding tissue. Eight weeks later, ewith the prolongation of implantation time, the macrophages decreased, and fibroblast morphology gradually became normal. In general, the inflammatory response was particularly light for all four hydrogels implanted in the rabbit subcutaneous. Additionally, after eight weeks, the inflammation disappeared, and the surrounding tissue looked the same as normal skin tissue. The degradation rate of these hydrogels themselves was all very slow, and coupled with no inflammatory response to stimulate the degradation, it became slower, which in turn relieved the inflammatory response.

### 3.14. In Vivo Studies in Rabbit Animal Model

#### 3.14.1. Observation of Wound Contraction and Healing

**Figure 17:**
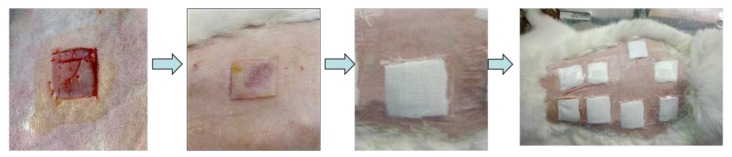
Process of full-thickness defect wounds preparation and the implantation of hydrogels.

**Figure 18:**
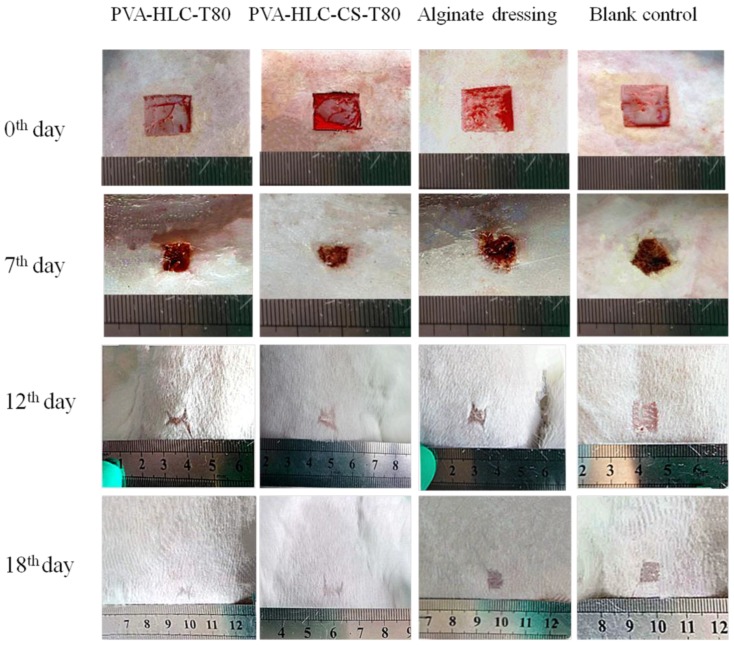
Change of full-thickness wound size after different period of time.

**Figure 19:**
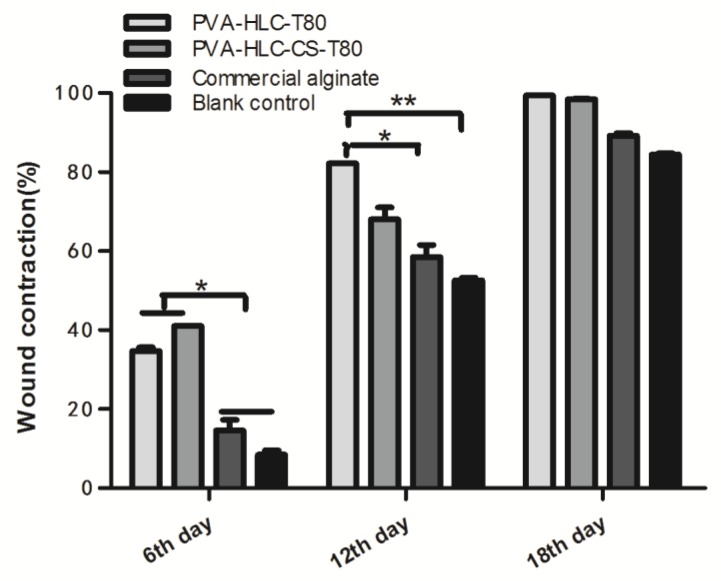
Contraction ratio of different hydrogel treated wounds after different periods of time. Where “*” and “**” represent the *p*-value < 0.05 and *p*-value < 0.01, respectively.

The wound healing performance of the hydrogels was further investigated by in vivo testing (Figure 17). The contraction ratio of the wounds in different groups was evaluated (Figure 18 and Figure 19). On the sixth, 12th and 18th days, the wound contraction for hydrogel PVA-HLC-T80 and PVA-HLC-CS-T80 groups had the advantage over the two control groups (both the commercial alginate dressing (Suprasorb A) and blank control group). On the sixth day, the hydrogel PVA-HLC-CS-T80 treated wound showed the smallest area (contracted 40.83%) among all groups, followed by the PVA-HLC-T80 treated wound group, which contracted 34.91% (*p* < 0.01). The commercial alginate dressing group showed a relatively small contraction (14.60%) and the contraction ratio of the blank wounds was only 8.48%. On the 12th day, the wound contraction ratio was similar to that of the sixth day. PVA-HLC-T80 group (82.25%), which was slightly better than the hydrogel PVA-HLC-CS-T80 group, had an approximate 40% lead than the commercial control (58.60%) groups (*p* < 0.01), and the blank control groups retained the least contraction where the ratio was only 52.66%. After 18 days, PVA-HLC-T80 was still in a leading position and contracted more than 99% and PVA-HLC-CS-T80 treated group contracted 98.5%, which still had a 16.7% lead over the two control groups (*p* < 0.01). Furthermore, wounds for the hydrogel PVA-HLC-T80 and PVA-HLC-CS-T80 groups were observed to have a closure on day 18 while wounds in the commercial dressing and blank control group still showed a different area of wounds. These data revealed that the wounds of the two hydrogel-treated groups recovered faster and healed earlier than the control groups, therefore demonstrating that the two hydrogels significantly promoted the recovery of full-thickness defect wounds.

Wound healing is a complex process that can be affected by many factors [36]. Thus, dressings with various properties that can enhance wound healing process are highly desirable. Considering the wound recovery rate, when compared with two control groups, PVA-HLC-T80 and PVA-HLC-CS-T80 groups had been in a leading position from the sixth day to the 18th day, perhaps due to the strong water absorbing capacity, large porosity, suitable water vapor permeability, excellent bacterial barrier activity and good hemostatic activity of these two hydrogels working together, which would contribute to accelerating the wound healing process.

#### 3.14.2. Histomorphological Evaluation of Wound Regeneration

**Figure 20:**
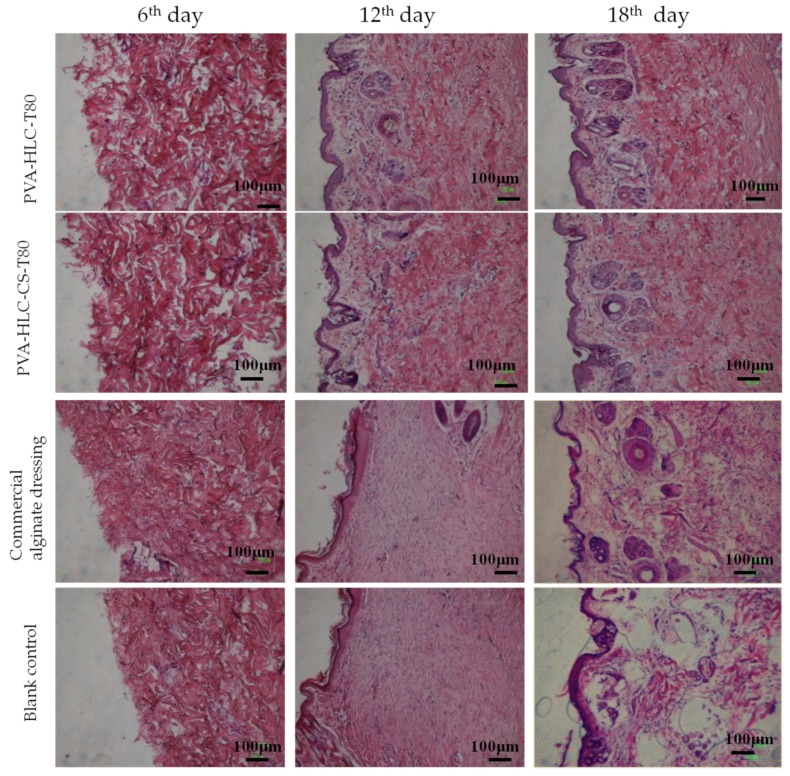
H&E photos of different hydrogel treated wounds after different periods of time.

The above results were further substantiated by histological analysis (Figure 20) of the specimens collected. Specimen sections were stained using hematoxylin and eosin staining. Wound regeneration was evaluated by observing the re-epithelization, fibroblast immigration, connective tissue synthesis, infiltration of inflammatory cells and collagen production.

On the sixth day, inflammatory cells appeared in all four groups, however, when compared with the commercial alginate dressing and blank control group, inflammatory cell infiltration was partly suppressed for the hydrogel PVA-HLC-T80 and PVA-HLC-CS-T80 hydrogels. Hemostasis of PVA-HLC-T80 and PVA-HLC-CS-T80 could rapidly stop blood bleeding. On the 12th day, when compared to the irregular structure seen in the two control groups, the PVA-HLC-T80 and PVA-HLC-CS-T80 hydrogel groups showed higher regularity of both the epithelium and connective tissue with more fibroblasts, and even a few hair follicles, which might be attributed to the synergistic effect of the multifunctions such as bacterial barriers activity and breathability, and of the hydrogels promoted cellular activities (such as proliferation and migration) including fibroblasts, keratinocytes, nerve, and muscle [37,38]. At 18th day, the wounds in the commercial dressing group formed a small number of basic structures of epithelium, dermism and a few hair follicles, but the re-epithelization and connective tissue arrangement were still not as regular and complete as the wounds in the PVA-HLC-T80 and PVA-HLC-CS-T80 groups. The blank control group retained an irregular structure with some collagen fibers distributed in disorder. In contrast, the tissue in the PVA-HLC-T80 and PVA-HLC-CS-T80 hydrogels groups exhibited excellent wound healing for better re-epithelization process, connective tissue remodeling and arrangement, more regular structure, and the formation of more hair follicles as the multifunctional properties of the hydrogel dressings promoted the wound healing process.

**Figure 21:**
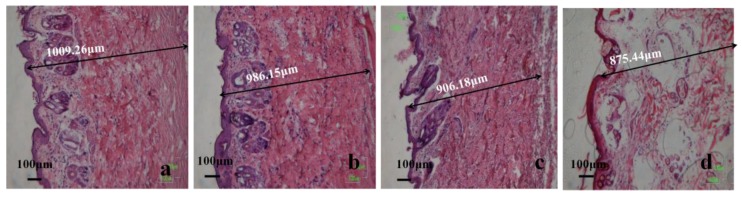
Granulation tissue of different hydrogel treated wounds after 18 days, (**a**–**d**) represent granulation tissue of PVA-HLC-T80, PVA-HLC-CS-T80, commercial dressing and blank control group respectively.

Granulation tissue in all groups showed differences between thickness and morphology (Figure 21). The granulation tissue in the PVA-HLC-T80 and PVA-HLC-CS-T80 hydrogel groups were 1009.26 μm and 986.15 μm, respectively, which was at least 80 μm thicker than that of the two control groups. Granulation tissue consists of accumulated extracellular matrix, fibroblasts, and other cells [39]. The wound healing process would benefit from the formation of a better granulation tissue with more accumulated extracellular matrixes, fibroblasts [40]. The in vivo study indicated that the hydrogel PVA-HLC-T80 and PVA-HLC-CS-T80 dressings improved wound healing through promoting the formation of granulation tissue.

#### 3.14.3. TEM Analysis

**Figure 22:**
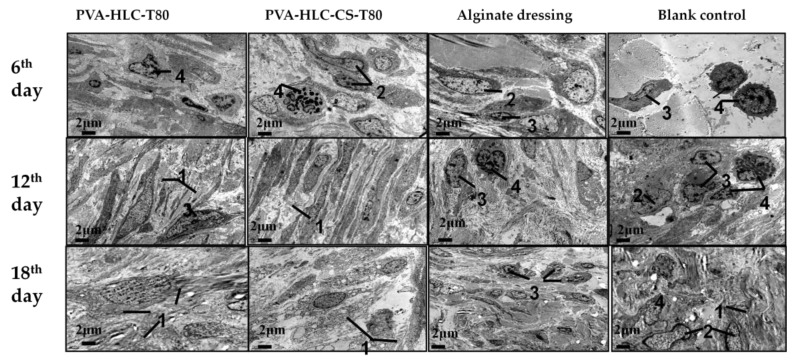
TEM photos of different hydrogel treated wounds after different periods of time, 1, 2, 3 and 4 represent collagen fibers, macrophage, lymphocyte and neutrophile granulocyte respectively.

TEM analysis (Figure 22) was used to monitor the changes in inflammation response during the recovery of full-thickness defects on rabbits. On the sixth day, four groups of wounds were exposed to different degrees of inflammation infiltration, where the rank of inflammation from heavy to light was as follows: blank control group, commercial alginate dressing group, PVA-HLC-T80, and PVA-HLC-CS-T80. On the 12th day, the inflammation had been greatly relieved, except for the blank control group with moderate inflammation. Finally, On the 18th day, the inflammation of the PVA-HLC-T80 and PVA-HLC-CS-T80 hydrogel groups almost disappeared, while the blank control group still revealed some inflammation cells. Moreover, the arrangement of cells in the two control groups was chaotic rather than neatly arranged like in the PVA-HLC-T80 and PVA-HLC-CS-T80 groups, which was similar to normal skin tissue.

## 4. Conclusions

We developed a series of soft, flexible, porous, translucent, breathable, non-adhesive hydrogel-based wound dressings, and demonstrated that they had good in vivo histocompatibility, moreover, they enhanced the wound healing process in a full-thickness skin defect model. These hydrogels were prepared by simply mixing PVA solution, HLC solution and CMCS solution in a certain proportion and adding Tween80 as a pore-forming agent, followed by two freez-thawing cycles, and finally removing the Tween80 by immersing these hydrogels in ultrapure water for three days. It is worth noting that the PVA-HLC-T80 and PVA-HLC-CS-T80 hydrogels showed a suitable moisture vapor transmission rate, outstanding hemostatic performance, and bacterial barrier activity which are beneficial in promoting the wound healing process, and also exhibited excellent mechanical properties, pore size, porosity, swelling ratio, biocompatibility, and histocompatibility. Furthermore, these two hydrogels remained soft after lyophilization, and greatly increased collagen deposition and granulation tissue formation compared to the commercial alginate dressing and blank control group by relieving the inflammation response and providing a comfortable environment for wounds. These results indicated that the combined effects of various functions of the two hydrogels obviously promoted full-thickness skin wound healing when compared to the commercial dressing, indicating their potential as dressings for skin wound healing.

## Figures and Tables

**Table 1:** Specific composition of different hydrogels.

Hydrogels	Content of Different Components (wt %)			
	PVA	HLC	CS	T80
PVA-HLC	6.67	1.67		
PVA-HLC-T80	6.67	1.67		2.4
PVA-HLC-CS	5.55	1.11	0.55	
PVA-HLC-CS-T80	5.55	1.11	0.55	2.4

**Table 2:** Density and porosity of different hydrogels.

The Hydrogels	Standards (mm)	Density (g·cm^−3^)	Porosity
PVA-HLC	38 × 38 × 3	0.1260 ± 0.002	39.88% ± 0.079
PVA-HLC-T80	38 × 38 × 3	0.0478 ± 0.010 **	85.01% ± 0.571 **
PVA-HLC-CS	38 × 38 × 3	0.1098 ± 0.008	38.20% ± 0.077
PVA-HLC-CS-T80	38 × 38 × 3	0.0534 ± 0.005 **	82.56% ± 0.093 **

PVA: Poly(vinyl alcohol). HLC: Human-like collagen. CS: Chitosan. T80: Tween80. “**” represent the *p*-value < 0.01, respectively.

**Table 3:** Water vapor transmission rate of different hydrogels.

Sample	Water Vapor Transmission Rate (g·m^−2^·d^−1^)
PVA-HLC	923.2 ± 7.1668 **
PVA-HLC-T80	2398.4 ± 3.1355 **
PVA-HLC-CS	921.1 ± 3.3391 **
PVA-HLC-CS-T80	2887.4 ± 4.3629 **
Control	4102.5 ± 2.5176

PVA: Poly(vinyl alcohol). HLC: Human-like collagen. CS: Chitosan. T80: Tween80. Where “**” represent the *p*-value < 0.01, respectively.

## Supplementary Materials

The supplementary materials are available online at www.mdpi.com/2073-4360/9/12/727/s1.
