# Supplementary material for: Preparation and Characterization of Breathable Hemostatic Hydrogel Dressings and Determination of Their Effects on Full-Thickness Defects
Source: Polymers (Basel). 2017 Dec 18;9(12):727. doi: 10.3390/polym9120727 (PMC6418977; doi:10.3390/polym9120727)
Supplement: Supplementary file 1 [file polymers-09-00727-s001.pdf]

Supplementary Materials:

Materials and Methods

S1: Synthesis of the hydrogels.

PVA pre-gel solution(10wt%) was prepared by dissolving 5g of PVA powder in 50ml of pure water at a temperature of 90℃ over a period of 2h. HLC pre-gel solution (5wt%) and CMCS pre-gel solution(2.5wt%) were prepared by dissolving 2.5g of HLC and 1.25g of CMCS in 50ml of pure water respectively. The preparation of Poly (vinyl alcohol)- human collagen(PVA-HLC) hydrogel was carried out by mixing PVA and HLC solution at a volume ratio of 2:1, then decanting the mixture into a customized square mold (with a square length of 40mm) and freezing for 20 hours at -20℃, then thawing for 4h at room temperature, this process was repeated 2 times. PVA-HLC-T80 hydrogel was prepared by the same process except that Tween 80 was added to the mixed solution at a volume ratio of 1:25 before pouring into the mold. For PVA-HLC-CS and PVA-HLC-CS-T80, the volume ratio of PVA, HLC and CS solution was 5:2:2, and Tween 80 was added as above described. The obtained hydrogels were then soaked in ultrapure water for 3 days with magnetic stirring to remove Tween80, the water was refreshed every two hours.

S2 Tensile property of the hydrogels

The hydrogels were cut into total length of 5.5 cm, breadth of 1.5 cm and for the gauge length of 2 cm. The samples were equilibrated with 0.1 M PBS (pH 7.4), and the elasticity was investigated by applying a load of 200 g at a cross-head speed of 10 mm/min. The ultimate tensile strength of the material was determined from the stress (kPa) versus strain (%) curve and the elongation length at break were also recorded.

S3 **Moisture vapor transmission rate.**

Take a measuring cup, which is corrosion resistance, impermeable and airtight. Add enough water to the cup so that the gap between the liquid level and the placed sample was 5±1 mm, seal the cup with the hydrogel dressing(with a thickness of 1.5~2 mm), the effective area of the sample is measured($57.2cm^2$), the total weight of the cup, sample and distilled water is weighed and recorded as W1.Then the equipment was placed in a drying oven or incubator. The same measuring cup, exposure in the air, was taken as control. 5 parallels were made for each sample. After 18h to 24 hours, removed the equipment from the oven or incubator, recorded the test time (T), immediately re-weighing the equipment, recorded as W2.

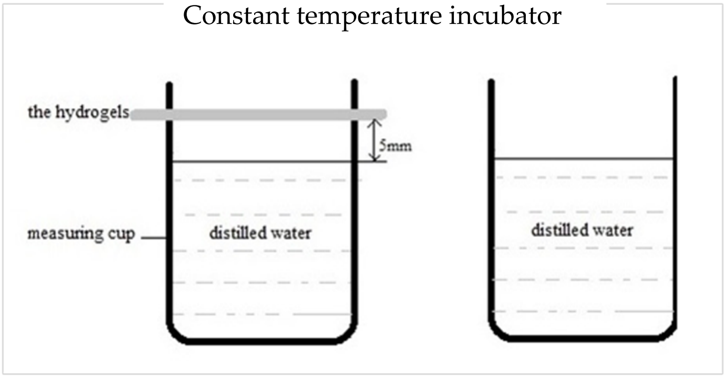

Fig. S1 The device to determine MVTR of the hydrogels. Figure in the left represents treatment group and the right one is control group.

S4 *In vitro* degradation rate

After weighed and sterilized by Co60 irradiation (24 h, 10 kGy), the dried gel samples were immersed in tubes containing 4ml of fresh SBF, and the tubes were kept static at 37 °C in an incubator. The samples were withdrawn from the SBF solution and then rinsed with ultra-pure water three times after soaking for different times. The dry weight was measured after lyophilization . The weight residual rate was calculated as:

$$WR = (W0 - W1)/W0 \ * 100\%$$

where W_0 and W_1 are the weights of the gel before and after soaking, respectively.

S5 *In vitro* hemolytic activity test

To prepare extract, the hydrogels were sterilized by Co60 irradiation, cut into small pieces and placed in tubes with fresh culture medium added at 0.1 mL/g at 37 ± 0.5 °C for 72 h, the whole process should be operated in the clean bench. The distilled water was taken as positive control and normal saline as negative control.

Specific test process: 5ml of blood was taken from healthy rabbit ear artery into a anticoagulant tube, then shake the tube quickly until the solution mixed evenly. 4ml of this anticoagulant blood was taken out and 5ml of 0.9% normal saline solution was added to prepare fresh dilution of rabbit blood. Then take 5ml of the four extracts, distilled water and normal saline solution respectively, add 0.1ml of the rabbit blood dilution to each tube and slowly mixed them evenly , then put the tubes to a 37 ℃ constant temperature water bath for 1h. After the water bath, centrifuge all the tubes at 4℃ (1500 rpm, 5 min) and carefully aspirate the supernatant. Their absorbance at 545 nm was measured with a spectrophotometer.

S6 Bacterial barrier properties

Staphylococcus aureus or E.coli were lawn cultured on a RODAC contact plate filled with nutrient agar medium. After incubation for 24 hours, the sterilized hydrogel sheets were placed on the surface of the plate, then another RODAC contact plate with nutrient agar medium was placed on the hydrogel samples and suppressed by a weight to make constant pressure on the materials . After incubation for 24 h, 48 h and 72 h, the RODAC contact plate on the top was removed and cultured at 30 ℃ for 24 h to observe whether there was any colony present.

S7 Hemostatic property

A bleeding ear artery rabbit model was employed and all animal studies were performed in compliance with guidelines set by national regulations and were approved by the local animal experiments ethical committee. The rabbit was anesthetized by injecting 3 wt% pentobarbital sodium（1ml/kg）and fixed on a surgical corkboard. The hair on back ear was shaved and the ear was sterilized by 75% alcohol. A pre-weighted filter paper on a polyurethane film was placed beneath the ear. A 1 cm × 1 cm wound including the ear artery was created, and the pre-weighted dressing was applied over the blooding area after free hemorrhage for 5 s and constant pressure (200 g) was exerted. The dressing was removed and weighted until blood was absolutely coagulated and the hemostatic time was recorded. Total blood loss was also evaluated by the difference value between the weights of dressing and filter paper before and after the hemostatic assay.

S8 Cytotoxicity testing

The hydrogels were sterilized by Co60 irradiation and then placed into tubes with fresh culture medium added at 0.1 mL/g, cultured at 37 ± 0.5 °C for 72 h to achieve hydrogel extracts. L929 cells were cultured at a density of 1.0 × 10^4 cells/mL on 96-well plates (100 μL/well) in a CO_2 (5%) incubator at 37 °C. After incubation for 24 h, the extracts were added to 96-well plates (100 μL/well) in a CO2 (5%) incubator at 37 °C. After incubation for 24 h, 72 h and 120 h, 10 μL of MTT solution was added to each well, the cultures were incubated at 37 °C for 4 h. Post incubation media containing MTT was removed, and the purple formazan crystals formed were dissolved by incubating the hyrogel in 1.5 mL of dimethyl sulfoxide (DMSO) for 15–20 min at RT with constant shaking. The absorbance of the solution was measured at 490 nm.

S9 *In vitro* biocompatibility

Fibroblast (L929) cell lines were cultured in 1640 media supplemented with 1% penicillinstreptomycin and 10% FBS in an incubator at 37 °C, 5% CO_2. The hydrogels (1~2 mm thickness and 10 mm diameter) were sterilized by Co60 irradiation (72 h, 10 kGy) and then they were washed 2–3 times with 1× PBS with 10–15 min incubation for each wash. Finally, the hydrogels were incubated in cell culture media for 24 h. To test the biocompatibility of the hydrogel, 1 × 10^5 of cells were seeded on each hydrogel sample. The media was refreshed every day.

MTT assay.

Cell viability was measured at regular time intervals by MTT assay. The hydrogels were sterilized by Co60 irradiation and then placed into tubes with fresh culture medium added at 0.1 mL/g, cultured at 37 ± 0.5 °C for 72 h to achieve hydrogel extracts. L929 cells were cultured at a density of 1.0 × 10^4 cells/mL on 96-well plates (100 μL/well) in a CO_2 (5%) incubator at 37 °C. After incubation for 24 h, the extracts were added to 96-well plates (100 μL/well) in a CO2 (5%) incubator at 37 °C. After incubation for 24 h, 72 h and 120 h, 10 μL of MTT solution was added to each well, the cultures were incubated at 37 °C for 4 h. Post incubation media containing MTT was removed, and the purple formazan crystals formed were dissolved by incubating the hyrogel in 1.5 mL of dimethyl sulfoxide (DMSO) for 15–20 min at RT with constant shaking. The absorbance of the solution was measured at 490 nm.

Microscopic Analysis.

The cells were allowed to grow for a period of 7 days in the hydrogel. These were analyzed by fluorescent staining. 1mm thick sections were selected and stained with AO/EB staining solution followed by detection with inverted fluorescence microscope.

S10 *In vivo* histocompatibility evaluation

All experiments were performed in compliance with the relevant laws and institutional guidelines, and conducted with the approval of the Institute Animal Ethics Committee. This study was supported by Shaanxi Key Laboratory of Degradable Biomedical Materials and Northwest University. The histocompatibility of hydrogels implanted into the body was measured at an immune system response level by hematoxylin and eosin(H&E) staining and transmission electron microscopy (TEM) analysis.

Implantation of hydrogels

The biocompatibility of the PVA-HLC,PVA-HLC-Tween80, PVA-HLC-CS,PVA-HLC-CS-Tween80 was assessed *in vivo* by implanting the shaved and disinfected hydrogels into rabbits. All of the rabbits were quarantined and were allowed free access to food and water, but were not given antibiotics for a week before surgery. The animals were raised in a relative humidity of 50–60%, a controlled temperature of 20–22 °C and a 12 h light-dark cycle. Dorsal hairs of rabbits were removed by depilation so that a naked region was obtained for the operation. The rabbits' backs were implanted 8 hydrogel sheets(0.5cm* 0.5cm), each of the four hydrogel dressings was repeated 2 times. After 2, 4, 6 and 8 weeks, the rabbits were depilated again and sacrificed. The gross appearance of implanted sites was photographed with a digital camera(Nikon TE2000-U), and the gel color, redness, edema, fester, vessels, and fiber capsule were assessed. The implant/tissue constructs were harvested and cut in half; one half was used for H&E staining and the other half for TEM.

H&E staining and TEM analysis.

In this analysis, the implant/tissue constructs harvested from the rabbits were immediately fixed in 10% neutral buffered formalin and left overnight. The constructs were rinsed with 0.1 M PBS (pH 7.4) for several minutes to remove residual formalin, then successively placed in a series of ethanol baths from70% to 100% ethanol before immersion in a mixture of ethanol and xylene (1:1 v/v) and xylene for 30 min each to ensure that the tissues were transparent. Next, the constructs were immersed in liquid wax overnight until the tissues were filled, then carefully embedded in epoxy resin. The blocks were sliced on a Leica RM2016 diamond saw microtome (Leica Instruments Ltd., Germany) with a blade thickness of 5 μm and assembled on coated slides.

For TEM analysis, the ultrathin sections (50 to 70 nm)were stained with 4% uranyl acetate and 0.5% lead citrate and observed by transmission electron microscopy (TEM) (HITACHI, H-600, Japan).

S11 *In Vivo* Studies in Rabbit Animal Model.

3-month old healthy female white New Zealand rabbits weighing 2−3 kg were used for experiments.

Surgical Procedure.

Before starting the surgery, rabbits were anaesthetized by giving intravenous injection with 3% pentobarbital (20mg/kg). Thereafter, an electric shaver was used to remove hair from the dorsal area and skin was sterilized using 70% ethanol. According to the principle that least number of animals were used，eight full thickness square defects were created (1.5cm x 1.5cm) on each rabbit and take every two symmetrical wounds as a group. The first two groups of wound were treated with PVA-HLC-Tween80 hydrogel and PVA-HLC-CS-Tween80 hydrogel，respectively. For comparison, the third group was treated with commercially available alginate dressings and the last group was left untreated (control). Each of these dressings was implanted on ten wounds for each time point (6th , 12th and 18th day after surgery). After dressings implantation, the wound area was covered with cotton gauze which was fixed by polyurethane film. After surgery, rabbits were housed individually in the cages. To evaluate repair, rabbits were sacrificed by giving intravenous injection of air after the 6th , 12th and 18th day of recovery. Specimens having whole wound area were collected for further analysis.

Macroscopic and Histological Evaluation.

After sacrificing the rabbits, the skin samples of the wound site were collected and fixed in 10% formalin saline. Further, samples were dehydrated by treating them with ethanol gradient. Thereafter, they were embedded in paraffin. Sections of 5 μm were cut and mounted on glass slides. For histological analysis, sections prepared were stained with hematoxylin and eosin. Samples of all the animals were analyzed for wound healing. There were 2 wounds per rabbit and 5 rabbits per condition making it 10 wounds per time point per condition. For each parameter, entire histological samples were analyzed with 10 fields taken under consideration. For comparison, the histology of normal rabbit skin was also performed.

Transmission electron microscope analysis.

To determine if the different treatments given generate any inflammatory response in rabbits, TEM analysis was done. For this, the ultrathin sections (50 to 70 nm)were stained with 4% uranyl acetate and 0.5% lead citrate and observed by transmission electron microscopy (TEM) (HITACHI, H-600, Japan).

The hydrogels were sterilized by Co60 irradiation and then placed into tubes with fresh culture medium added at 0.1 mL/g, cultured at 37 ± 0.5 °C for 72 h to achieve hydrogel extracts. L929 cells were cultured at a density of 1.0×10^4 cells/mL on 96-well plates (100 μL/well) in a CO_2 (5%) incubator at 37 °C. After incubation for 24 h, the extracts were added to 96-well plates (100 μL/well) in a CO2 (5%) incubator at 37 °C. After incubation for 24 h, 72 h and 120 h, 10 μL of MTT solution was added to each well, the cultures were incubated at 37 °C for 4 h. Post incubation media containing MTT was removed, and the purple formazan crystals formed were dissolved by incubating the hyrogel in 1.5 mL of dimethyl sulfoxide (DMSO) for 15–20 min at RT with constant shaking. The absorbance of the solution was measured at 490 nm.
